# Efficacy and safety of pelvic floor magnetic stimulation combined with mirabegron in female patients with refractory overactive bladder: a prospective study

**DOI:** 10.3389/fnins.2024.1373375

**Published:** 2024-04-10

**Authors:** Ping Liang, Qing-lai Tang, Tao Lin, Zheng-kun Tang, Fa-de Liu, Xing-zhu Zhou, Rong-zhen Tao

**Affiliations:** ^1^Department of General Surgery, The Second Hospital of Nanjing, Affiliated Hospital to Nanjing University of Chinese Medicine, Nanjing, Jiangsu, China; ^2^Department of Urology, The Affiliated Jiangning Hospital of Nanjing Medical University, Nanjing, Jiangsu, China; ^3^Department of Urology, Liaocheng People’s Hospital, Liaocheng, Shandong, China; ^4^Department of Clinical Medicine, Kangda College of Nanjing Medical University, Lianyungang, Jiangsu, China

**Keywords:** refractory overactive bladder, pelvic floor magnetic stimulation, mirabegron, combination therapy, incontinence

## Abstract

**Objective:**

To observe the efficacy and safety of pelvic floor magnetic stimulation (PFMS) combined with mirabegron in female patients with refractory overactive bladder (OAB) symptoms.

**Patients and methods:**

A total of 160 female patients with refractory OAB symptoms were prospectively randomized into two groups. Eighty cases in the combination group accepted PFMS and mirabegron therapy and 80 cases as control only accepted mirabegron therapy (The clinical trial registry number: ChiCTR2200070171). The lower urinary tract symptoms, OAB questionnaire (OAB-q) health-related quality of life (HRQol), symptom bother score and OABSS between two groups were compared at the 1st, 2nd and 4th week ends.

**Results:**

All of 160 patients were randomly assigned to two groups, of which 80 patients were included in the combination group and 80 in the mirabegron group. The incidences of LUTS, including urgency, frequent urination, and incontinence episodes, in the 2nd week and the 4th week after combination treatment were significantly lower than those in the mirabegron group (*p* < 0.05). The incidence of drug-related adverse events between two groups was similar, and there was no statistically significant difference (*p* > 0.05). With respect to secondary variables, the OAB-q HRQol score in the combination group was statistically superior in comparison with that in the mirabegron group between the 2nd week and the 4th week (*p* < 0.05). This was consistent with the primary outcome. Meanwhile, from the second to fourth week, the OAB-q symptom bother score and OABSS in the combination group were both lower than in the mirabegron group (*p* < 0.05).

**Conclusion:**

Combination therapy of PFMS and mirabegron demonstrated significant improvements over mirabegron monotherapy in reducing refractory OAB symptoms for female patients, and providing a higher quality of life without increasing bothersome adverse effects.

**Clinical Trial Registration:**

https://www.chictr.org.cn/, ChiCTR-INR-22013524.

## 1 Introduction

As everyone knows, overactive bladder (OAB), defined by the International Association for Urology, was a syndrome characterized by urgency of urination, often accompanied by symptoms of frequent urination and nocturia, with or without urgent urinary incontinence, without urinary tract infections or other clear pathological changes (Haylen et al., 2010). According to data published in 2011, the overall prevalence of OAB in China was 6.0%, with 5.9% in males and 6.0% in females. The overall prevalence of OAB increases significantly with age.

OAB can have a significant impact on the quality of patients’ life (Wang et al., 2011). There were many treatment methods for OAB, mainly including behavioral therapy, drug therapy, invasive therapy (botulinum toxin injection, nerve stimulation therapy), etc. (Malde et al., 2018). Mirabegron, the first and only selective β3-adrenergic receptor agonist agent, has appeared as an emerging drug class for the treatment of urinary incontinence, urgency, and frequency caused by OAB (Igawa and Michel, 2013). Meanwhile, our team’s previous study showed that mirabegron acts through a different mechanism to relieve OAB symptoms with fewer adverse effects (Tang et al., 2022). Neuroregulation inhibits detrusor muscle contraction by stimulating peripheral nerves to induce inhibitory neural reflexes. Therefore, pelvic floor magnetic stimulation (PFMS) of the S3 nerve root can acutely inhibit idiopathic detrusor instability and plays a significant role in the treatment of OAB. Choe et al. found that 27 patients (56.3%) were cured which OAB treated with magnetic stimulation alone compared to baseline at 2 weeks (Ho et al., 2007). The maximum urine output did not show a significant change, but the average urine output significantly increased after stimulation.

However, there were still a considerable number of patients who experienced the above treatment, achieved poor results and accompanied by frequent urinary urgency and abnormal urination behavior, seriously affecting their daily life and work. The American Society of Urology defined refractory OAB as: poor relief of OAB symptoms after long-term behavioral training, or failure to receive an anticholinergic medication for 6–12 weeks (including poor symptom relief or intolerable adverse reactions) (De Wachter et al., 2019). In order to improve clinical efficacy, many scholars have begun to try comprehensive therapies for refractory OAB. Currently, there are few studies on the use of mirabegron or PFMS in female patients with refractory OAB symptoms. Hence, we conducted this prospective, randomized trial to explore new treatment models and assess the effectiveness and safety of PFMS combined with mirabegron for refractory OAB.

## 2 Methods

### 2.1 Patients

**Figure 1:**
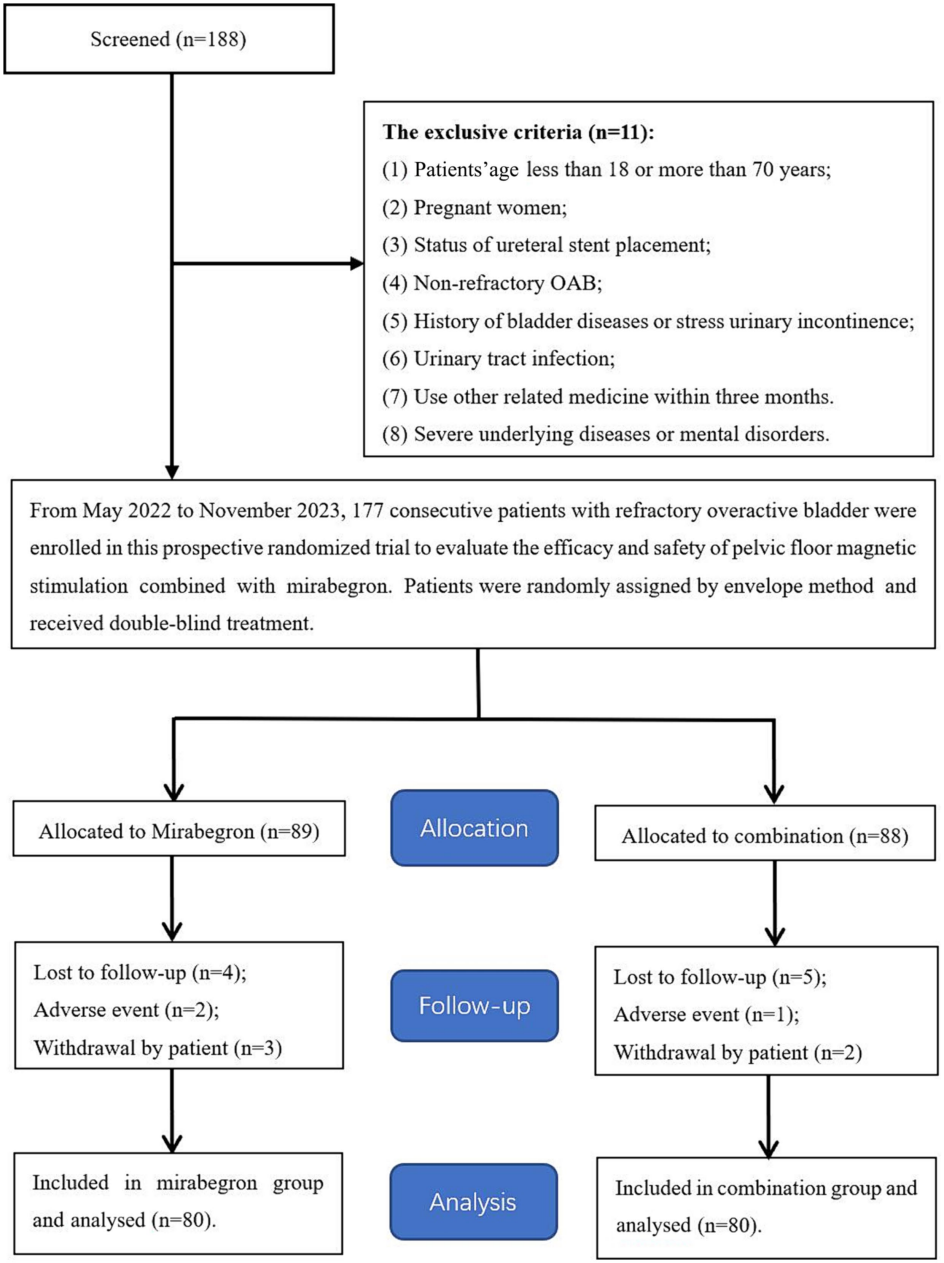
Flowchart for case selection.

From May 2022 to November 2023, eligible female patients with refractory OAB symptoms who were referred to our institute were considered for this study. These patients were divided into the mirabegron group (including 80 patients) and the combination group (including 80 patients) under applying strict inclusion criteria and randomly assigning the patients by the envelope method (Figure 1). The pre-treatment evaluation data of each participant included general information, medical history, physical examination, laboratory examination and imaging examination. The study was approved by the clinical research ethics committee of the Affiliated Jiangning Hospital of the Nanjing Medical University (ethics approval number: 202200137). Written informed consent was obtained from all participants.

In this prospective study, we evaluated the effectiveness and safety of mirabegron and combination of PFMS and mirabegron in relieving refractory OAB symptoms, using the overactive bladder questionnaire (OAB-q) health-related quality of life (HRQol), symptom bother score and overactive bladder syndrome score (OABSS).

### 2.2 Study procedure

**Figure 2:**
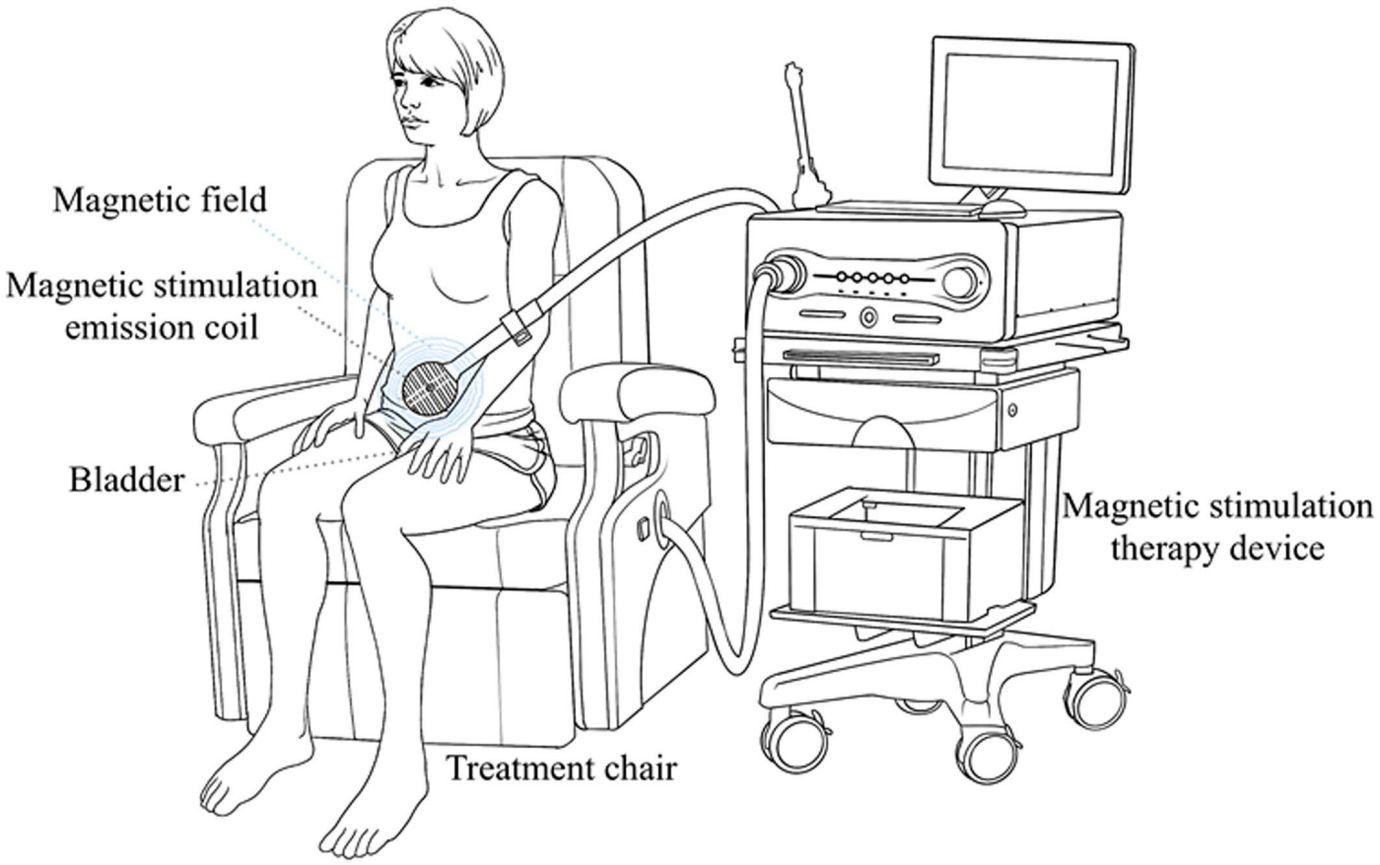
Diagram for pelvic floor magnetic stimulation.

The patients in both groups in this study were independent and randomized. Mirabegron 50 mg once a day was given to the patients in the mirabegron group. The other patients in the combination group received mirabegron 50 mg once daily and PFMS twice a week. Magnetic stimulation therapy was performed on patients using Magneuro 30F magnetic stimulator (Weisi, Nanjing). The patient did not need to undress and sited directly on the treatment chair, and chose the treatment mode for overactive bladder (Figure 2). Adjust the intensity of stimulation based on the patient’s subjective perception, with an appropriate intensity of 5% increase after the patient had a sense of anal contraction. The specific parameters of treatment device were set to a stimulation frequency of 10 Hz, a stimulation time of 4 s, a rest time of 6 s, and a treatment time of 20 min.

All patients were advised to drink enough water to maintain a daily urine volume more than 1,500 mL. Each patient was also asked to complete a real-time questionnaire to report potential drug-related adverse events, and clinical symptoms related to urgency, frequent urination, and incontinence episodes. At the same time, relevant questionnaires were completed by the patients during the follow-up period. The patient can contact the attending doctor at all times if there were any problems related to treatment. If at the end of the follow-up period, the patient’s discomfort symptoms have not been relieved, or the remission effect was not satisfactory, relevant further auxiliary treatment will be carried out.

### 2.3 Follow-up

There was no significant difference at the time of diagnosis and treatment between the two groups. The patients reported the drug-related adverse events each week. Lower urinary tract symptoms (LUTS) at different times was considered as the primary outcome of the study. The secondary end points were OAB-q HRQol score, symptom bother score and OABSS. Treatment failure was defined as the lack of improvement or relief of OAB symptom.

### 2.4 Statistical analysis

SPSS v.32.0 for Windows (IBM Corp., Armonk, NY, USA) was used to perform statistical analysis. Continuous variables were presented as mean ± standard deviation. Patient demographics, follow-up time and clinical outcomes between the two groups were compared using independent samples t test; Chi-squared test was used to compare other clinical characteristics between the two groups. A *p* < 0.05 was considered significant.

## 3 Results

In this study, 160 patients were randomly assigned to two groups: 80 patients in the mirabegron group and 80 patients in the combination group. The patients’ demographics and clinical characteristics were shown in Table 1. The mean age was 47.3 years in the mirabegron group and 48.2 years in the combination group, respectively, and no significant difference was found in the patient age between the two groups (*p* = 0.406). After further study based on the type of OAB, there was still no obvious difference between the two groups. In addition, there was no significant difference between the two groups in body mass index, hypertension and diabetes history, or received previous treatment with an anticholinergic medication (All *p* > 0.05).

**Table 1:** Comparisons of patients’ demographics and clinical characteristics between two groups.

Variables, mean ± SD or *n* (%)	Mirabegron group (*n* = 80)	Combination GROUP (*n* = 80)	*p* value
Age, year	47.3 ± 6.1	48.2 ± 7.5	0.406
BMI, kg/m^2^	24.7 ± 3.3	25.1 ± 2.9	0.417
Hypertension history			
No	51 (63.7)	48 (60.0)	–
Yes	29 (36.3)	32 (40.0)	0.625
Diabetes history			
No	39 (48.7)	36 (45.0)	–
Yes	41 (51.3)	44 (55.0)	0.635
Received previous treatment with an anticholinergic medication			
No	22 (27.5)	17 (21.2)	–
Yes	58 (72.5)	63 (78.8)	0.357
Type of OAB			
Urgency incontinence only	55 (68.7)	58 (72.5)	0.603
Mixed incontinence	23 (28.8)	19 (23.8)	0.472
Frequency/urgency without incontinence	2 (2.5)	3 (3.7)	0.650

BMI = body mass index; SD = standard deviation.

Differences in the clinical outcomes between the two groups were shown in Tables 2, 3. The incidences of LUTS, including urgency, frequent urination, and incontinence episodes, in the 2nd week and the 4th week after combination treatment were significantly lower than those in the mirabegron group (*p* < 0.05). And the gap between the two groups was getting bigger and bigger. In terms of drug- related adverse events, there was no statistically significant difference between two groups (*p* > 0.05). No other serious complications were noted in this study.

**Table 2:** Comparisons of clinical outcomes between two groups.

Variables, *n* (%)	Mirabegron group (*n* = 80)	Combination group (*n* = 80)	*p* value
Urgency			
1st week	77 (96.2)	73 (91.2)	0.191
2nd week	61 (76.2)	46 (57.5)	0.012*
4th week	35 (43.7)	19 (23.7)	0.007**
Frequent urination			
1st week	76 (95.0)	71 (88.7)	0.148
2nd week	63 (78.7)	50 (62.5)	0.024*
4th week	39 (48.7)	23 (28.7)	0.009**
Incontinence episodes			
1st week	71 (88.7)	69 (86.2)	0.633
2nd week	56 (70.0)	43 (53.7)	0.034*
4th week	28 (35.0)	12 (15.0)	0.003**
Drug related adverse events	5 (6.2)	4 (5.0)	0.732
Urinary tract infection	1 (1.2)	1 (2.5)	–
Tachycardia	2 (2.5)	2 (2.5)	–
Hypertension	2 (2.5)	1 (1.2)	–

SD = standard deviation; *p < 0.05, **p < 0.01.

**Table 3:** Comparisons of OAB-symptom score between two groups.

Variables, mean ± SD or *n* (%)	Mirabegron group (*n* = 80)	Combination group (*n* = 80)	*p* value
OAB-q HRQol score			
Baseline	59.4 ± 5.3	58.6 ± 4.9	0.323
1st week	62.7 ± 4.1	63.9 ± 5.2	0.107
2nd week	77.5 ± 4.6	79.1 ± 4.4	0.026*
4th week	86.3 ± 5.1	88.7 ± 4.8	0.003**
OAB-q symptom bother score			
Baseline	59.2 ± 3.8	58.3 ± 4.1	0.152
1st week	48.3 ± 4.1	47.3 ± 3.9	0.116
2nd week	37.5 ± 3.9	36.2 ± 3.2	0.022*
4th week	25.2 ± 3.3	23.6 ± 3.7	0.004**
OABSS			
Baseline	9.2 ± 2.3	9.4 ± 1.9	0.550
1st week	8.1 ± 2.0	7.7 ± 1.5	0.154
2nd week	6.6 ± 2.1	5.8 ± 2.2	0.020*
4th week	3.2 ± 1.7	2.4 ± 1.6	0.003**

SD = standard deviation; OAB-q = overactive bladder questionnaire; HRQol = health-related quality of life; OABSS = overactive bladder syndrome score. *p < 0.05, **p < 0.01.

With respect to secondary variables, the OAB-q HRQol score in the combination group was statistically superior in comparison with that in the mirabegron group between the 2nd week and the 4th week (*p* < 0.05). This was consistent with the primary outcome. Meanwhile, from the second to fourth week, the OAB-q symptom bother score and OABSS in the combination group were both lower than in the mirabegron group (*p* < 0.05).

## 4 Discussion

The pelvic floor muscles interact with the bladder and urethra through nerves, playing a role in maintaining complete bladder emptying and inhibiting contraction feedback. Women often suffer from pelvic floor muscle damage due to childbirth and other gynecological surgeries, or the imbalance of feedback loop caused by changes in hormone secretion levels as they age. The inhibition pathways of pelvic floor muscle groups are limited, leading to bladder instability and urinary incontinence (Rogers et al., 2018). With the aging population, more and more elderly female patients are facing serious difficulties due to OAB (Chughtai et al., 2023). However, with the improvement of modern Chinese women’s cultural level and awakening of awareness, more and more patients are pursuing and yearning for a high-quality life (Paiva et al., 2017). Therefore, the treatment demand market for OAB is very large.

Currently, various studies have proposed the pathogenesis of OAB, but there are mainly three hypothesis theories. The firstly, neurogenic hypothesis suggests that increased sensitivity of sensory nerve endings, increased excitability of spinal cord reflexes, or pathological changes in descending inhibitory pathways lead to increased voluntary contractile activity of the detrusor muscle (De Groat, 1997). Then the myogenic hypothesis suggests that ultrastructural changes in the detrusor muscle lead to autonomous contraction of muscle cells and enhanced intercellular electrical activity (Haferkamp et al., 2003). Lastly, the epithelial origin hypothesis showed that changes of receptors or neurotransmitters in bladder epithelial cells lead to abnormalities in the integration and transmission pathways of bladder sensory signals (Birder and Andersson, 2013). However, the above hypotheses cannot perfectly explain the pathogenesis of OAB, and the disease cannot be completely cured in clinical treatment. Dagdeviren et al. found that sympathetic nervous system excitation, oxidative stress, and metabolic related pathological changes caused an increase in serum neurotrophic factor levels, which was closely related to the onset of OAB (Dagdeviren and Cengiz, 2018). Liu et al. (2013) found that the level of C-reactive protein, neurotrophic factor, IL-1β, IL-6, IL-8, and TNF-α in patients with refractory OAB was significantly increases, and they believed that chronic inflammation of the bladder mediated changes in peripheral and central nervous system transmission pathways, making clinical treatment more complex. We speculated that the pathogenesis of refractory OAB may be the result of the interaction between the aforementioned hypotheses and theories.

OAB and urgent urinary incontinence are often related to excessive excitation of the detrusor muscle. Magnetic stimulation may inhibit the overactivity of the detrusor muscle by stimulating the anal rectal branch of the pelvic floor nerve and pudendal nerve, or by stimulating the spinal cord afferent nerve root (S3) it can also activate the inhibitory pathway (Shalom et al., 2014; Corcos et al., 2017). In 1992, Bemelmans et al. applied magnetic stimulation of the cauda equina nerve to detect motor evoked potentials in the bladder and pelvic floor (Bemelmans et al., 1992). The application of magnetic stimulation of the sacral nerve root could activate the inhibitory pathway of the spinal cord, suppress the impulses of the detrusor motor neurons, and thus suppress the urinary reflex or unstable contraction and hyperreflexia of the detrusor muscle. Further, Khedr et al. (2011) reported that they applied lumbar sacral magnetic stimulation to treat patients with refractory neurogenic urinary dysfunction caused by lumbar sacral nerve injury, and found that 15 Hz stimulation can improve symptoms of urgency and urinary incontinence. The magnetic field has strong penetrating power and can excite deep tissues which difficult to reach by electrical stimulation. The magnetic stimulation emission coil can move flexibly outside the body, and the stimulation site can be adjusted according to the treatment of diseases. The operation is simple and non-invasive. By adjusting different parameters and stimulation time, it can treat different types of urinary dysfunction diseases, which has important clinical application value (de Wachter et al., 2020).

The research on drug intervention is mainly based on the distribution of receptors in the bladder. The nervous system mainly affects the activity of bladder smooth muscle through the transmission of neurotransmitters (Maldonado-Avila et al., 2016). Adrenoceptors are widely expressed in the bladder and ureteral tissue based on our team’s previous studies (Tang et al., 2021, 2022). Mirabegron, a type of β3-adrenoceptor agonists, has appeared as an emerging drug class for the treatment of urinary incontinence, urgency, and frequency caused by OAB (Igawa and Michel, 2013). The potential role of β3-adrenoceptor agonists in OAB treatment is based on the findings that the human bladder expresses β3-adrenoceptors, and that these β3-adrenoceptors are predominant. Previous study have shown that monotherapy with mirabegron can significantly reduce the frequency of nocturia and urinary incontinence, and compared to non-selective M receptor blockers, mirabegron has a more significant effect on improving nocturia (Christopher, 2014).

Considering the complexity of the pathogenesis of refractory OAB and the difficulty of clinical treatment, exploring appropriate treatment options remained a current research hotspot. Based on this, this prospective, randomized trial was conducted to explore new treatment models and assess the effectiveness and safety of PFMS combined with mirabegron for refractory OAB. All patients in the two groups were diagnosed with refractory OAB and completed follow-up. Our current study showed that the incidences of LUTS, including urgency, frequent urination, and incontinence episodes, in the 2nd week and the 4th week after combination treatment were significantly lower than those in the mirabegron group (*p* < 0.05). The main acting mechanisms demonstrated that combination treatment can stimulate bladder detrusor relaxation and promote urine storage, resulting in an increased bladder volume and prolonged urination interval without affecting bladder emptying. In terms of drug-related adverse events, the incidence in the two groups was similar, and there was no statistically significant difference (*p* > 0.05). No other serious complications were noted in this study. With respect to symptom score, the OAB-q HRQol score in the combination group was statistically superior in comparison with that in the mirabegron group between the 2nd week and the 4th week (*p* < 0.05). Secondly, from the second to fourth week, the OAB-q symptom bother score was higher in the mirabegron group than in the combination group (*p* < 0.05). Lastly, the OABSS was also lower in the combination group than that in the mirabegron group between the 2nd week and the 4th week (*p* < 0.05).

However, this study has some limitations. The time for follow-up was short and it may have affected the outcome. Furthermore, we only counted the relevant symptoms through the patient’s main complaint, which may have subjective deviation. Finally, the study was based on a single center with a small sample size, and there may be a certain amount of sampling error. Therefore, large-scale multicenter prospective studies are required to further prove the above conclusions. It is likely that the ideal procedure will be formulated through a long period of clinical application and observation.

## 5 Conclusion

Combination therapy of PFMS and mirabegron demonstrated significant improvements over mirabegron monotherapy in reducing refractory OAB symptoms for female patients, and providing a higher quality of life without increasing bothersome adverse effects. Combination therapy should be strongly considered for female patients who complain of OAB symptoms. In our opinion, this method is safe and reproducible in clinical practice. However, future prospective clinical studies should include objective physiological measures such as urine flow rate as well as the inclusion of a PFMS alone treatment group to further prove the above conclusions.

## Data availability statement

The datasets presented in this study can be found in online repositories. The names of the repository/repositories and accession number(s) can be found in the article/supplementary material.

## Ethics statement

The studies involving humans were approved by the clinical research ethics committee of the Affiliated Jiangning Hospital of the Nanjing Medical University. The studies were conducted in accordance with the local legislation and institutional requirements. The participants provided their written informed consent to participate in this study.

## Author contributions

PL: Writing – review & editing, Conceptualization. Q-lT: Writing – review & editing. TL: Writing – original draft. Z-kT: Writing – original draft. F-dL: Writing – original draft, Data curation. X-zZ: Writing – review & editing, Formal analysis. R-zT: Writing – review & editing, Conceptualization.

## References

[ref1] Bemelmans B. L. van Kerrebroeck P. E. V. Notermans S. L. Wijkstra H. Debruyne F. M. J. (1992). Motor evoked potentials from the bladder on magnetic stimulation of the cauda equina: a new technique for investigation of autonomic bladder innervations. J. Urol. 147, 658–661. doi: 10.1016/S0022-5347(17)37339-1, PMID: 1538450

[ref2] Birder L. Andersson K. E. (2013). Urothelial signaling. Physiol. Reviews 93, 653–680. doi: 10.1152/physrev.00030.2012, PMID: 23589830 PMC3768101

[ref3] Christopher C. (2014). Mirabegron the first β3-adrenoceptor agonist for overactive bladder (OAB): a summary of the phase III studies. BJU Int. 113, 847–848. doi: 10.1111/bju.12773 24905658

[ref4] Chughtai B. Ricker C. N. Boldt R. J. Elterman D. (2023). Real-world onabotulinumtoxinA treatment patterns in patients with overactive bladder. Neurourol. Urodyn. 43, 396–406. doi: 10.1002/nau.25370, PMID: 38149719

[ref5] Corcos J. Przydacz M. Campeau L. Gray G. Hickling D. Honeine C.. (2017). CUA guideline on adult overactive bladder. Can. Urol. Assoc. J. 11, E142–E173. doi: 10.5489/cuaj.4586, PMID: 28503229 PMC5426936

[ref6] Dagdeviren H. Cengiz H. (2018). Association between metabolic syndrome and serum nerve growth factor levels in women with overactive bladder. Gynecol. Obstet. Investig. 83, 140–144. doi: 10.1159/000477170, PMID: 28637031

[ref7] De Groat W. C. (1997). A neurologic basis for the overactive bladder. Urology 50, 36–52. doi: 10.1016/S0090-4295(97)00587-6 9426749

[ref8] De Wachter S. Benson K. D. Dmochowski R. R. Rovner E. S. Versi E. Miller L. E.. (2019). Six-month results of selective bladder denervation in women with refractory overactive bladder. J. Urol. 201, 573–580. doi: 10.1016/j.juro.2018.09.043. PMID: 30240691, PMID: 30240691

[ref9] de Wachter S. Knowles C. H. Elterman D. S. Kennelly M. J. Lehur P. A. Matzel K. E.. (2020). New technologies and applications in sacral neuromodulation: an update. Adv. Ther. 37, 637–643. doi: 10.1007/s12325-019-01205-z, PMID: 31875299 PMC7004424

[ref10] Haferkamp A. Dorsam J. Elbadawi A. (2003). Ultrastructural diagnosis of neuropathic detrusor overactivity: validation of a common myogenic mechanism. Adv. Exp. Med. Biol. 539, 281–291. doi: 10.1007/978-1-4419-8889-8_20, PMID: 15088911

[ref11] Haylen B. T. de Ridder D. Freeman R. M. Swift S. E. Berghmans B. Lee J.. (2010). An international Urogynecological association (IUGA)/international continence society (ICS) joint report on the terminology for female pelvic floor dysfunction. Neurourol. Urodyn. 29, 4–20. doi: 10.1002/nau.20798, PMID: 19941278

[ref12] Ho C. J. Myung-Soo C. Kyu-Sung L. (2007). Symptom change in women with overactive bladder after extracorporeal magnetic stimulation: a prospective trial. Int. Urogynecol. J. Pelvic Floor Dysfunct. 18, 875–880. doi: 10.1007/s00192-006-0261-0 17136485

[ref13] Igawa Y. Michel M. C. (2013). Pharmacological profile of b3-adrenoceptor agonists inclinical development for the treatment of overactive bladder syndrome. Naunyn Schmiedeberg's Arch. Pharmacol. 386, 177–183. doi: 10.1007/s00210-012-0824-1, PMID: 23263450

[ref14] Khedr E. M. Alkady E. A. el-Hammady D. H. Khalifa F. A. M. bin-Humam S. (2011). Repetitive lumbosacral nerve magnetic stimulation improves bladder dysfunction due to lumbosacral nerve injury: a pilot randomized controlled study. Neurorehabil. Neural Repair. 25, 570–576. doi: 10.1177/1545968311400091, PMID: 21411715

[ref15] Liu H. T. Jiang Y. H. Kuo H. C. (2013). Increased serum adipokines implicate chronic inflammation in the pathogenesis of overactive bladder syndrome refractory to antimuscarinic therapy [J]. PLoS One 8, 1–5. doi: 10.1371/journal.pone.0076706 PMC3788120 24098552

[ref16] Malde S. Fry C. Schurch B. Marcelissen T. Averbeck M. Digesu A.. (2018). What is the exact working mechanism of botulinum toxin a and sacral nerve stimulation in the treatment of overactive bladder/detrusor overactivity? ICI-RS 2017. Neurourol. Urodyn. 37, S108–S116. doi: 10.1002/nau.23552, PMID: 30133790

[ref17] Maldonado-Avila M. Garduno-Arteaga L. Jungfermann-Guzman R. Manzanilla-Garcia H. A. Rosas-Nava E. Procuna-Hernandez N.. (2016). Efficacy of tamsulosin, oxybutynin, and their combination in the control of double-j stent related lower urinary tract symptoms[J]. Int. Braz J Urol 42, 487–493. doi: 10.1590/S1677-5538.IBJU.2015.0186, PMID: 27286111 PMC4920565

[ref18] Paiva L. L. Ferla L. Darski C. Catarino B. M. Ramos J. G. L. (2017). Pelvic floor muscle training in groups versus individual or home treatment of women with urinary incontinence: systematic review and meta-analysis. Int. Urogynecol. J. Pelvic Floor Dysfunct. 28, 351–359. doi: 10.1007/s00192-016-3133-2 27613622

[ref19] Rogers R. G. Pauls R. N. Thakar R. Morin M. Kuhn A. Petri E.. (2018). An international Urogynecological association (IUGA)/international continence society (ICS) joint report on the terminology for the assessment of sexual health of women with pelvic floor dysfunction. Neurourol. Urodyn. 37, 1220–1240. doi: 10.1002/nau.23508, PMID: 29441607

[ref20] Shalom D. F. Pillalamarri N. Xue X. Kohn N. Lind L. R. Winkler H. A.. (2014). Sacral nerve stimulation reduces elevated urinary nerve growth factor levels in women with symptomatic detrusor overactivity. Am. J. Obstet. Gynecol. 211, 561.e1–561.e5. doi: 10.1016/j.ajog.2014.07.007, PMID: 25019486

[ref21] Tang Q.-l. Wang D.-j. Zhou S. Tao R.-z. (2021). Mirabegron in medical expulsive therapy for distal ureteral stones: a prospective, randomized, controlled study. World. J. Urol. 39, 4465–4470. doi: 10.1007/s00345-021-03772-9, PMID: 34241685

[ref22] Tang Q.-l. Zhou S. Liu Y.-q. Wu J. Tao R.-z. (2022). Efficacy and safety of combination of mirabegron and solifenacin in patients with double-J stent related overactive bladder: a prospective study. Sci. Rep. 12:18844. doi: 10.1038/s41598-022-23795-5 36344629 PMC9640653

[ref23] Wang Y. Xu K. Hu H. Zhang X. Wang X. Na Y.. (2011). Prevalence, risk factors, and impact on health related quality of life of overactive bladder in China. Neurourol. Urodyn. 30, 1448–1455. doi: 10.1002/nau.21072. PMID: 21826714, PMID: 21826714

